# Multisystemic Increment of Cortical Thickness in Congenital Blind Children

**DOI:** 10.1093/texcom/tgaa071

**Published:** 2020-10-09

**Authors:** Alberto Inuggi, Anna Pichiecchio, Benedetta Ciacchini, Sabrina Signorini, Federica Morelli, Monica Gori

**Affiliations:** Unit for Visually Impaired People (U-VIP), Istituto Italiano di Tecnologia, 16152 Genova, Italy; Department of Brain and Behavioural Neuroscience, University of Pavia, 27100 Pavia, Italy; Neuroradiology Department, IRCCS Mondino Foundation, 27100 Pavia, Italy; Radiology Department, University of Pavia, 27100 Pavia, Italy; Department of Translational Medicine, Università del Piemonte Orientale, 28100 Novara, Italy; Centre of Child Neuro-Ophthalmology, Child Neuropsychiatry Unit, IRCCS Mondino Foundation, 27100 Pavia, Italy; Department of Brain and Behavioural Neuroscience, University of Pavia, 27100 Pavia, Italy; Centre of Child Neuro-Ophthalmology, Child Neuropsychiatry Unit, IRCCS Mondino Foundation, 27100 Pavia, Italy; Unit for Visually Impaired People (U-VIP), Istituto Italiano di Tecnologia, 16152 Genova, Italy

**Keywords:** children, congenital blindness, cortical thickness, development, magnetic resonance imaging (MRI)

## Abstract

It has been shown that the total or partial lack of visual experience is associated with a plastic reorganization at the brain level, more prominent in congenital blind. Cortical thickness (CT) studies, to date involving only adult subjects, showed that only congenital blind have a thicker cortex than age-matched sighted population while late blind do not. This was explained as a deviation from the physiological mechanism of initial neural growth followed by a pruning mechanism that, in congenital blind children, might be reduced by their visual deprivation, thus determining a thicker cortex. Since those studies involved only adults, it is unknown when these changes may appear and whether they are related to impairment degree. To address this question, we compared the CT among 28 children, from 2 to 12 years, with congenital visual impairments of different degree and an age-matched sighted population. Vertex-wise analysis showed that blind children, but not low vision one, had a thicker cortical surface in few clusters located in occipital, superior parietal, anterior-cingular, orbito-frontal, and mesial precentral regions. Our data suggest that the effect of visual impairment on determining thicker cortex is an early phenomenon, is multisystemic, and occurs only when blindness is almost complete.

## Introduction

A complete or severe congenital lack of visual experience may have a negative impact on children’s motor, cognitive, and relational development. Visually impaired (VI) children tend to manifest impairments in the motor domain (Levtzion-Korach et al. 2000) but also in several spatial aspects, specifically in auditory and proprioceptive localization (Cappagli and Gori 2016; Vercillo et al. 2016; Cappagli et al. 2017), haptic orientation discrimination (Gori et al. 2010), and reach on sound (Elisa et al. 2002). Those deficits may reflect themselves into an altered brain development (Merabet and Pascual-Leone 2010), being its trajectory still under debate. It is well known that early brain development is based on a primary synaptogenesis mechanism that generates almost all of the neurons of the mature cortex within the first 7 months after birth (Goswami 2004). What follows is a complex mechanism of neuronal plasticity, which is modulated by individual sensorial experiences and sensory–motor interactions (Hubener 2014). In the visual cortex, the synaptic density reaches a peak of around 150% of the adult counterpart between 4 and 12 months of age. Later on, it gradually reduces itself down to the adult level (Johnson 1997) through a synaptic regression conventionally called “pruning.” This process would complete a few years later, around either 5 (Johnson 1997) or 11 years (Huttenlocher and de Courten 1987). While the initial synaptogenesis-driven increment of synaptic density does not depend on visual experience (Winfield 1981; Bourgeois and Rakic 1996), it has been demonstrated that the following pruning is instead driven by vision (Stryker and Harris 1986; Bourgeois and Rakic 1996). Converging evidence suggests that cortical thickness (CT), the distance between the gray matter (GM)/white matter (WM) interface surface with pial 1 measured from *T*_1_-weighted anatomical magnetic resonance imaging (MRI) images, might represent a valid measure of such pruning. Since MRI-derived measures are merely our best current approximations and other factors, such as myelination and cortical morphology, modulate CT (Natu et al. 2019), “apparent cortical thickness” might be a more correct term (Walhovd et al. 2017). Nevertheless, CT proved to be a good candidate measure for detecting pathological (Sowell et al. 2008) and neurodevelopmental (Sowell et al. 2004; Shaw et al. 2006, 2007) changes, and it has been validated against manual measurement from in vivo or postmortem brain scans (Kuperberg et al. 2003; Salat et al. 2004). Several studies on blind adults have supported the idea that visual experience significantly modulates people’s CT. In blind adults, a consistent increase of CT, mainly located in primary visual areas (Jiang et al. 2009; Park et al. 2009; Voss and Zatorre 2012; Li et al. 2017) or widespread in frontal, cingular, pre and postcentral regions (Anurova et al. 2015), have indeed been reported. Although indirectly, it has been hypothesized that such thickening might be predominantly determined by a reduction of synaptic pruning (Uylings 1998; Jiang et al. 2009; Li et al. 2017) rather than by a cross-modal reorganization following visual deprivation (Voss and Zatorre 2012). What resulted in being determinant for thickness modulation was the onset of the visual deprivation. Indeed, when populations of blind individuals were compared with controls, CT increments were evident only in congenital or early but not in late blind individuals (Jiang et al. 2009; Li et al. 2017), suggesting the existence of a critical period for brain development. Accordingly, the presence of such a critical period was also confirmed by consistent literature of behavioral and neurophysiological studies (Gori et al. 2014; Vercillo et al. 2016; Cappagli et al. 2017), reporting how blindness onset profoundly shapes individual performance (Wan et al. 2010; Voss 2013, 2016). Presently, the cortex profile of early blind individuals could only be assessed ex post, through studies involving only adult subjects and has not been investigated directly during childhood. In the present study, we aim instead at measuring congenital blind children’s CT changes while this occur in childhood, without the bias of long-term phenomena such as aging and cross-modal plastic reorganization (Voss and Zatorre 2012; Anurova et al. 2015). Both of these phenomena occur until adulthood and may have an opposite effect on thickness: While age induces a progressive thinning of the cortex, cross-modal reorganization, but also specific training and intense use, may induce focal and selective thickenings of the cortex, particularly in the sensory-deprived areas (Kupers and Ptito 2014; Renier et al. 2014). The overall net contribution of aging and cross-modal reorganization may thus be partly unpredictable and affect the correct interpretation of studies involving blind adults only. Here we hypothesize that the cortex profile of early blind individuals should start deviating from sighted children since the first years of life. To address our hypothesis, we investigated (a) whether a CT difference was measurable since childhood, (b) whether it evolved with age, (c) whether it depended on the severity of visual impairment, and (d) whether it was limited to V1 or multisystemic. To this goal, we measured the CT in 2 populations of VI children and compared them with an age-matched population of sighted children.

## Methods

### Subjects Enrollment

This is a retrospective study obtained from anamnestic, clinical, neuro-ophthalmological, and MRI data recorded during the period from September 2009 to August 2016. All data were collected under the ethical principles for medical research involving human subjects stated in the Declaration of Helsinki. Participants’ parents signed an informed agreement stating that their children’s data, once anonymized, might have been used for research purposes. Participants were included according to an iterative process aimed at grouping VI children according to their visual acuity and define a group of normally sighted controls (NSC) children with the most similar age and gender profiles. This process also had to minimize the bias of having recorded subjects with different *T*_1_ sequences. Enrollment started from the Centre of Child Neuro-Ophthalmology that defined a list of VI children fulfilling the following clinical criteria.

#### Inclusion Criteria

- Children with congenital visual impairment due to involvement of anterior visual pathways or oculomotor system not related to central disturbances.
- Impairment ranging from total blindness to visual acuity below 3/10.

Such value was determined in compliance with the Italian law, which defines a 3/10 visual acuity at a 3 m distance as the cutoff to define the low vision (LV), determined with a standardized test (e.g., with optotype). Blindness is defined as 3 m distance visual acuity <0.05/10 (G.U. Serie Generale, n. 93 del 21 Aprile 2001).

#### Exclusion Criteria

- Any clinical sign of central nervous system involvement.

For each subject in the list, the Neuroradiology Department verified the presence and good quality of the corresponding MRI data, excluded those patients that showed any sign of central lesions or abnormalities on MRI evaluation, and took note of the *T*_1_ sequence they were recorded with.

#### NSC Recruitment Criteria

NSC subjects were searched among children with normal MRI that underwent the exam to investigate possible causes of behavioral disorders, language delay, or a specific hypotonia. We included those children whose age was within the VI group ages range, normal neurological examination, and without any abnormalities of visual functions. NSC group final composition was done minimizing age, gender, and MRI sequence differences with respect to the final VI group.

### Data Collection

#### Demographic and Clinical Data

Participants’ demographic, anamnestic, and clinical data were collected. The best-corrected visual acuity was evaluated, according to children’s age and cooperation, by means of either the Teller Acuity Cards (Teller et al. 1986) or an octotype for far or near distance in each eye.

#### MRI Data

All data were recorded in the Neuroradiology Department of IRCCS Mondino Foundation (Pavia, Italy). During brain MRI, complete stillness is an absolute requirement for adequate image quality. Anesthesia for MRI was necessary for 24 children (16 VI children and 8 NSC) aged <7 years, to both ensure immobility and reduce discomfort associated with the diagnostic procedure (Mongodi et al. 2019), whereas it was not required in the remaining, cooperative patients. Anesthesia was performed by a standard approach consisting of sevoflurane-based induction and maintenance. Whenever indicated, premedication with midazolam was administered intramuscularly 30 min before anesthesia (Mongodi et al. 2019). The MRI scans were acquired from 2 different MRI *T*_1_ sequences performed on the same 1.5 T Intera Master Nova Philips scanner with the following protocols:

Sequence #1:

Repetition time (TR)/echo time (TE)/flip angle = 25 ms/4.6 ms/30°, Number of excitation (Nex) = 1, matrix = 240 × 240 sagittal, in-plane resolution = 0.88 mm × 0.88 mm, and spacing between slices = 0.8.

Sequence #2:

TR/TE/flip angle = 8.5 ms/4 ms/8°, Nex = 1, matrix = 288 × 288 sagittal, in-plane resolution = 0.9 mm × 0.9 mm, spacing between slices = 1.2 mm.

**Figure 1:**
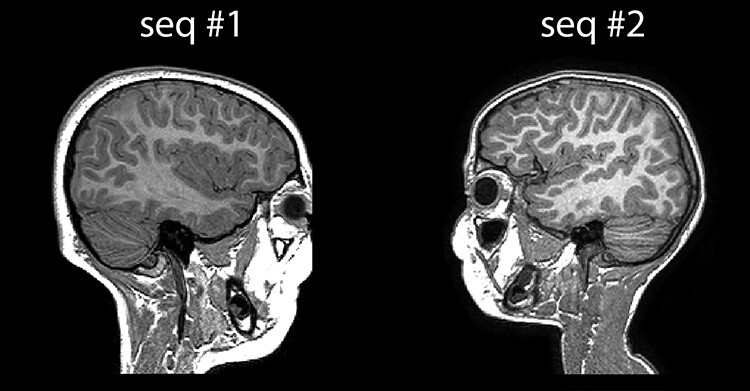
Examples of *T*_1_ images obtained with the 2 sequences.

An example of obtained scans is represented in Figure 1.

### MRI Processing

Three-dimensional *T*_1_-weighted MRI scans were converted to NIFTI format and resliced from sagittal to axial orientation. They were visually inspected, and their origin was set in correspondence of the anterior commissure. The following processes were carried out with the Computational Analysis Toolbox (CAT, version 12.6) within SPM12 using MATLAB (version 2017b). All images were normalized using an affine followed by nonlinear registration, corrected for bias field inhomogeneity, and then segmented into GM, WM, and cerebrospinal fluid components (Ashburner and Friston 2005). The Diffeomorphic Anatomic Registration Through Exponentiated Lie (DARTEL) algebra algorithm was used to normalize the segmented scans into a standard Montreal Neurological Institute (MNI) space (Klein et al. 2009) using 6 iterations. Compared with the conventional algorithm, the DARTEL approach can provide more precise spatial normalization to the template (Matsuda et al. 2012). As part of the modulation step, we performed a nonlinear deformation on the normalized segmented images with the CAT12 toolbox. This modulation provides a comparison of the absolute amounts of tissue corrected for individual differences in brain size (Cousijn et al. 2012). All segmented, modulated, and normalized GM and WM images were smoothed using 8-mm full-width-half-maximum (FWHM) Gaussian smoothing. Pediatric anatomical templates for both segmentation and normalization steps were created with the CerebroMatic Toolbox (Wilke et al. 2017) to improve overall reconstruction accuracy. The 2 templates were created specifying the same age and gender data of our population.

CT was evaluated according to the projection-based thickness method (Dahnke et al. 2013). The surface extraction pipeline used topology correction (Yotter et al. 2011a), spherical mapping (Yotter et al. 2011b), estimation of local surface complexity, and local gyrification (Luders et al. 2006). Finally, cortex surfaces were smoothed (FWHM = 15 mm) and resampled to a 32 k mesh compatible with the Human Connectome Project.

For each participant, we thus obtained a CT surface composed of the values of its 64 000 vertexes. In addition, the subjects’ mean CT (mCT) and total intracranial volume (TIV) values were calculated.

#### Data Quality

*T*_1_ data quality and homogeneity were calculated with the CAT software and used as 1 of the criteria applied to define the final group’s composition.

### Statistical Analysis

We tested our data to evaluate 2 distinct phenomena. First, since the 2 different *T*_1_ sequences might have introduced a bias within our analyses, we first evaluated the “sequence” effect over our measures. Second, we explored the effect of the “group” factor over CT. Both these analyses were done at the subject level (age, mCT, and TIV) and the vertex level (CT maps).

#### Subject Level

We anticipate that being subjects’ mCT not normally distributed and pretending to model the possible interaction between group and sequence, a nonparametric ordinal logistic regression (OLR) model (Cessie and Houwelingen 1992), suited for factorial analysis, was tested on mCT and TIV. OLR analysis was implemented through the “lrm” function of the Regression Modeling Strategies (rms) package (Frank E. Harrell Jr 2017) within R software [R Core Team (2017). R: A language and environment for statistical computing. R Foundation for Statistical Computing, Vienna, Austria]. Once discarded either the main effect or the interaction of the sequence, we ignored this factor and focused on the effect of group and age and their interaction on subjects’ mCT and TIV, again with an OLR model.

#### CT Maps

All vertex-wise analyses were done with the Stats tool of SPM package.

##### Sequence effect

Aware that merging data recorded with different sequences may induce an unpredictable effect on our results, we tried to be as conservative as possible, that is, to create an exclusion mask composed by all those vertices that could also be minimally affected by the sequence effect. Four different two-samples *t*-test, 1 considering the whole population (15 subjects recorded with sequence #1 and 31 with sequence #2), to increase the test power, and 3 within each population (sighted, LV, and blind), to may take into account possible interaction between “sequence” and “group,” were run. To increase the liberality of the test, that is, also marking those vertices that showed just a trend toward such effect, all the tests were not corrected for multiple comparisons (*P* < 0.005). The 4 thresholded maps underwent a logic “or” operation; to be included in the mask, and thus excluded from the results, it was just necessary that the vertex appeared in whichever 1 of the 4 analyses.

##### Group effect

A one-way analysis of variance (ANOVA), using default values and unequal data variance, was run to assess the “group” effect. Subjects’ age was included as a covariate and its main effect and interaction with “group” were also calculated. This model allows estimating the effect of age considering all children together and, since it includes 1 age-related regressor for each group, also evaluating differences between the slope of within-groups correlations. Results were corrected for multiple comparisons using the family-wise error (FWE) criterion with a *P* value <0.05. The exclusion mask created in the sequence-effect analysis was applied a posteriori only to validate the results. We reported only those vertices that did not appear in that exclusion mask. In this way, all the results were obtained without reducing the number of multiple comparisons and without preliminary excluding any vertexes from the analysis. As a further analysis, we repeated the ANOVA using only the subjects recorded with the most numerous sequences. Although this latter cohort was quite small, its results could fortify previous ones and understand the possible effect of mixing subjects recorded with different sequences.

#### Age Effect on Significant Clusters

In case vertices-wise analysis failed in finding an age effect on CT maps, a cluster-based analysis was run. For each cluster affected by the group factor, individual CT values were averaged and analyzed through a factorial (group and age) OLR model. When an age × group interaction was found, a nonparametric Spearman correlation was run between age and clusters’ mean values, separately for each group. Post hoc analyses of group effects were run with Bonferroni-corrected nonparametric Wilcoxon–Mann–Whitney tests.

#### Visual Acuity Analysis

The visual acuity at 3 m was the only performance measure that were collected and recorded in all the enrolled subjects. Nevertheless, a regression analysis looking for a significant correlation between visual acuity and thickness could be actually performed only in low-vision subjects (LVS) group. In fact, enrolled sighted children did neither report any visual disturbance nor wear any glasses and their visual acuity was not recorded and considered as 10. Blind children acuity at 3 m was always below 0.05 and equal to 0 in most of the cases. In both these cases, correlation analysis could not produce any valid results. A multiple regression analysis, including visual acuity and age regressors, was performed only in LVS group. Results were corrected for multiple comparisons using the FWE criterion with a *P* value <0.05. The exclusion mask was applied as in previous analyses. The regression analysis was replied at the cluster level, in each cluster affected by the group factor, with an age-corrected OLR model.

## Results

### Subjects

The starting list of VI children with MRI data and fulfilling clinical criteria included 44 subjects, with ages ranging from 0.39 years (142 days) to 12.42 years, recorded with 3 different MRI sequences. After having discarded subjects with excessive movements (#3), those recorded with a *T*_1_ sequence characterized by a bad GM/WM contrast (#7), and those that had problems with surface extraction (#2), we decided to remove 4 very young VI children (from 142 to 398 days) for the following reason: We did not have so many young NSC subjects. Hence, it would have been impossible to match the VI and NSC groups ages, and the CAT homogeneity evaluation tool marked them as possible outliers, discouraging their inclusion.

#### Visually Impaired 

Data from 28 VI children were included in the study. According to their visual acuity, 6 were totally blind (T-BS), 7 had minimal close-up visuals with no visual perception for far distance (F-BS), and the remaining 15 had LV (LVS) with residual vision for far distance (visual acuity at 3 m below 3/10). To allow statistical comparisons, the T-BS and F-BS groups were merged in the same blind subjects (BS) group. The implications of this forced choice will be discussed throughout the manuscript. Clinical characteristic of the VI group is summarized in Table 1. In both BS and LVS, the number of subjects recorded with the second sequence was double than those recorded with the first one. Accordingly, the same proportion was used in NSC.

**Table 1:** Visually impaired subjects

Subject	Gender	Age (years)	Diagnosis	Deficit	Sequence	Subject	Gender	Age (years)	Diagnosis	Deficit	Sequence
BS_01	F	2.1	CRD	PVD	1	LVS_01	M	8.1	OM	PVD	1
BS_02	F	3.8	CRD	PVD	1	LVS_02	F	7.9	CRD	PVD	2
BS_03	M	5.8	CRD	PVD	2	LVS_03	M	9.5	CRD	PVD	2
BS_04	M	12.3	CRD	PVD	2	LVS_04	F	7.3	NYS	AOM	2
BS_05	M	7.7	CRD	PVD	2	LVS_05	M	5.0	CRD	PVD	1
BS_06	M	10.9	CRD	PVD	2	LVS_06	M	3.4	CRD	PVD	1
BS_07	F	2.0	CRD	PVD	2	LVS_07	F	2.7	NYS	AOM	1
BS_08	M	6.3	CRD	PVD	2	LVS_08	F	3.2	OA	PVD	2
BS_09	M	2.1	CRD	PVD	1	LVS_09	F	3.7	ONH	PVD	2
BS_10	F	10.6	CRD	PVD	2	LVS_10	M	9.4	CRD	PVD	2
BS_11	F	6.8	CRD	PVD	2	LVS_11	M	8.2	CRD	PVD	2
BS_12	M	5.4	CAT	PVD	2	LVS_12	F	12.4	NYS, SM	AOM	2
BS_13	M	5.1	ONH	PVD	1	LVS_13	M	6.2	ONH	PVD	2
						LVS_14	M	9.7	ONH	PVD	2
						LVS_15	M	6.8	CID	AOM	1

Note: Left column: 13 blind subjects (BS). The first 6 are totally blind (T-BS), the following 7 are far blind (F-BS). Right column: 15 low-vision subjects (LVS). The Sequence column indicates the MRI sequence used. F, female; M, male; CRD, congenital retinal dystrophy; ONH, optic nerve hypoplasia; PVD, peripheral visual deficit; AOM, altered ocular movement; NYS, nystagmus; CID, congenital innervation dysgenesis syndrome; OA, ocular albinism; SM, severe myopia; OM, ocular malformation; CAT, cataract.

#### Normally Sighted Controls

Data from 18 NSC (10 male and 8 female) were included in the study. Their mean age was 6.9 ± 2.5 years (range: 2.2–12.1 years). In all, 12 were recorded with sequence #2 and 6 with sequence #1.

### Mean Values Distribution

Nonparametric tests were selected for the non-normality of mCT values (Shapiro test: *W* = 0.93785, *P* = 0.016). Although TIV (*W* = 0.9823, *P* = 0.7012) and age (*W* = 0.96975, *P* = 0.2711) were normally distributed, to preserve tests uniformity, they were analyzed with the same tests.

### The MRI Sequence Effect

#### Mean Values

No effect of either group (χ^2^ = 5.08, *P* = 0.27), sequence (χ^2^ = 8.23, *P* = 0.083) or their interaction (χ^2^ = 1.53, *P* = 0.46) was found on mCT by OLR analysis. The same was found for ITV (group: χ^2^ = 4.29, *P* = 0.36; sequence: χ^2^ = 6.79, *P* = 0.14; group × sequences: χ^2^ = 3.71, *P* = 0.15).

#### Thickness Maps

**Figure 2:**
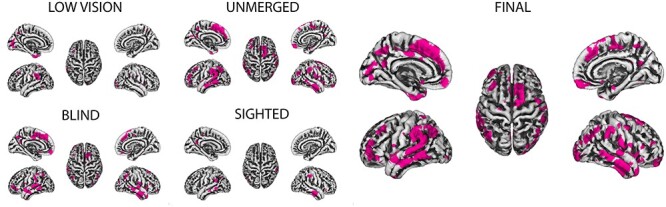
Exclusion mask. Left: Vertices are affected by sequence effect considering all subjects (unmerged) or each population separately. Right: Final mask composed by the union (logical “OR”) of the 4 maps on the left.

The 4 uncorrected two-samples *t*-test on the sequence effect revealed, respectively, considering all subjects or only those belonging to the 3 populations (blind, LV, and sighted), 5877, 4139, 1041, and 1002 vertices, which resulted significantly thicker in sequence #2 with respect to sequence #1. The resulting exclusion mask, created as the union among the above 4 results mask, was composed of 8695 vertices, which represents the 13.38% of the total vertexes number (64 984). Affected voxels are displayed in Figure 2.

### The Group Effect

#### Mean Values

**Figure 3:**
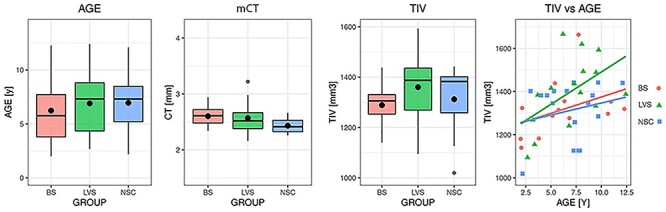
Distribution of mCT, TIV, and age across groups.

No effect of either “group” (χ^2^ = 7.53, *P* = 0.11), “age” (χ^2^ = 3.59, *P* = 0.30), or their interaction (χ^2^ = 2.48, *P* = 0.28) was found on mCT by OLR analysis. Analysis on ITV did not reveal either the effect of “group” (χ^2^ = 4.05, *P* = 0.39) or of “group × age” (χ^2^ = 1.39, *P* = 0.49), but showed a slight effect of age (χ2 = 7.93, *P* = 0.047) instead. The results are summarized in Table 2 and represented in Figure 3.

**Table 2:** Children age and mean structural scores

Groups	Age (years)	mCT (mm)	TIV (cm^3^)
NSC	6.9 ± 2.5	2.46 ± 0.12	1299 ± 150
LVS	6.9 ± 2.8	2.57 ± 0.27	1398 ± 163
BS	6.2 ± 3.4	2.60 ± 0.17	1308 ± 165

#### Thickness Maps

**Figure 4:**
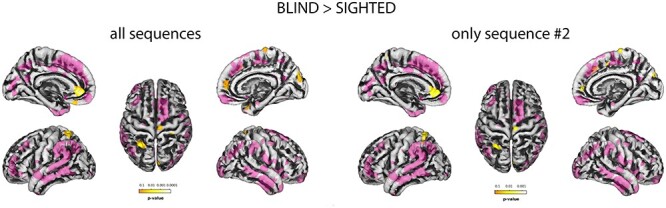
Group effect over cortical thickness, as revealed by two-samples *t*-test. Increased thickness in blind children with respect to sighted 1. Results were FWE corrected, *P* < 0.05. Left: Using all subjects. Right: Using only subjects recorded with sequence #2. The exclusion mask is overlaid in semitransparent violet.

Vertex-wise, one-way ANOVA of the group effect over CT revealed 9 clusters of different thickness. Post hoc analysis of such effect revealed that such differences were entirely due to thickening, exactly in the same regions, in BS cortex compared with NSC one. These clusters were located in the right medial occipital cortex (V2/V3) and bilateral anterior cingulate cortex (ACC), superior parietal cortex (SPC) and right lateral postcentral gyrus, temporal pole, infero-temporal cortex, and lateral orbito-frontal cortex (OFC). One cluster, located in mesial precentral gyrus, was found only in the post hoc analysis. One cluster, located in middle cingulate cortex (MNI: *x* = 5, *y* = 29, *z* = 34), overlapped with the exclusion mask and was not further considered. Since the results of the 2 analyses basically coincided, only the latter were summarized (Table 3) and displayed (Fig. 4, left). For completeness, the former was included in Supplementary Materials only (Supplementary Fig. S1 and Supplementary Table S1). Neither the interaction between “age” and “groups,” nor an effect of “age” over CT was found in any vertex.

**Table 3:** Effect of group (FWE-corrected one-way anova) over vertex-wise cortical thickness, considering subjects recorded with both sequences

Area	Cl. dim.	*P*	*Z*-score	*x*	*y*	*z*
L ACC	121	<0.001	5.37	−6	38	−3
R ACC	90	0.021	4.6	7	49	6
R OFC	128	<0.001	5.16	28	18	−24
R cuneal cortex (V2/V3)	36	0.011	4.76	6	−85	28
L SPC	95	0.001	4.94	−26	−55	66
R SPC	25	0.017	4.53	18	−48	70
R mesial precentral (M1)	37	0.01	4.54	5	−22	78
R lateral postcentral (S1)	19	0.022	4.87	31	−32	70
R temporal pole	48	0.006	4.6	28	11	−31
R ITC	16	0.025	4.6	41	−15	−31

Notes: Dim, number of vertices belonging to the cluster; ITC, inferior temporal cortex; L, left; R, right.

The ANOVA was repeated using only the 31 children recorded with sequence #2. This analysis could basically confirm the previous one. Only BS children showed a thickening in a few clusters with respect to NSC. The right postcentral, superior parietal, temporal pole, and infero-temporal gyrus (ITG) clusters disappeared, whereas the extensions of the remaining clusters reduced, together with a *P* value/*Z*-score generally increment/decrement. Two clusters partially overlapped with the exclusion mask and were not considered. One, located in middle cingulate cortex (MNI: *x* = 5, *y* = 29, *z* = 34), coincided with that found in previous analysis, the other was located in supplementary motor areas (MNI: *x* = 5, *y* = 1, *z* = 49). Only the 6 clusters resulting from both the analyses, summarized in Table 4 and represented in Figure 4 (right), were considered in the discussion. For completeness, group main effect results were included in Supplementary Materials only (Supplementary Fig. S2 and Supplementary Table S2).

**Table 4:** Effect of group (FWE-corrected one-way anova) over vertex-wise cortical thickness, considering only subjects recorded with sequence #2

Area	Dim.	*P*	*Z*-score	*x*	*y*	*z*
L ACC	116	0.027	4.84	−3	42	−7
R ACC	53	0.003	4.6	9	48	2
R OFC	23	0.015	4.58	26	17	−15
R cuneal cortex (V2/V3)	12	0.026	4.57	6	−85	34
L SPC	66	0.001	4.41	−21	−58	68
R mesial precentral (M1)	31	0.009	4.76	5	−21	78

Notes: Dim, number of vertices belonging to the cluster; L, left; R, right.

No significant differences emerged between LVS and the other 2 groups. Trying to assess whether LVS thickness pattern was closer to 1 of the 2 groups, uncorrected post hoc analysis was run. Results, shown in Supplementary Figure S2, revealed that no differences could be observable between blind and LV, whereas a trend versus a higher thickness was found in LVS compared with NSC in some of the regions already found thicker in blind versus sighted.

### Age × Group Effect on Significant Clusters

The “group × age” effect on CT was investigated only in the 6 clusters that resulted significantly affected by the “group” factor in both the vertices-wise analysis. The results are reported in Supplementary Table S3. All clusters mCT values were, as expected, affected by “group” main factor. Bonferroni-corrected post hoc Wilcoxon–Mann–Whitney tests confirmed a higher thickness in blind with respect to sighted in all clusters. The only cluster affected by “age” was the right V2/3 one. There a main effect of “age” (χ^2^ = 18.55, *P* = 0.0003) and an “age × group” interaction (χ^2^ = 7.22, *P* = 0.027) was found. Within groups, nonparametric, correlation between age and CT was negative and significant in NCS group (ϱ = −0.52, *P* = 0.026), showed a negative trend (ϱ = −0.48, *P* = 0.068) in LVS, and was absent in BS (*P* = 0.33).

### Correlation Between Visual Acuity at 3 m and Thickness

Thickness–acuity regression analysis in LVS group did not show any relation between the 2 measures, neither in vertices nor in clusters analyses.

## Discussion

There is converging evidence suggesting that early acquired or congenital visual deprivation alters brain development. Among structural modifications, CT has been consistently found thicker in congenitally blind adults V1 compared with sighted individuals (Jiang et al. 2009; Park et al. 2009; Voss and Zatorre 2012; Anurova et al. 2015; Li et al. 2017) and such thickening was dependent on blindness onset, being greatest comparing sighted against early blind individuals and gradually decreasing with later blindness onset ages (Li et al. 2017). This is the first work investigating brain thickness changes in blind children during development, comparing 2 different populations of congenitally VI children, from 2 to 12 years old, with an age-matched population of sighted children. This gave us the possibility to measure congenital blind children’s thickness changes while they actually occur, thus minimizing the bias of both age-related thinning and late, long-term, plastic reorganization occurring until adulthood, which affected all previous studies on the adult blind.

Results suggest that the previously reported cortical thickening with respect to sighted could be observed only in blind (BS) and not in LV children. These differences did not evolve with age, being evident already in our younger subjects and were local and multisystemic. While whole brain mCT was not affected by visual experience, differences emerged in several brain regions, such as in right mesial cuneal cortex (V2/V3), lateral OFC, mesial precentral region, in bilateral ACC, and left SPC.

**Figure 5:**
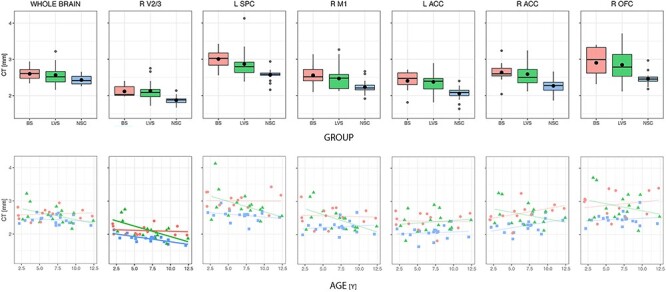
Age × group analysis within each cluster. Upper: Means across groups. Cortex resulted significantly thicker only in blind with respect to sighted. Lower: Correlation between age and CT. Only in right V2/3 cluster, a negative correlation was found between CT and age in sighted and a trend in LVS.

The main result of the present study was that the brain of congenitally blind children already showed that pattern of increased thickness found in congenital and early blind adults regions (Jiang et al. 2009; Park et al. 2009; Voss and Zatorre 2012; Anurova et al. 2015; Li et al. 2017). Blind thickening with respect to sighted did not evolve with age and was already present also in our younger blind children. This would imply that most of the thickening induced by visual impairment might have taken place in the very first years of children development. Since our cohort was small and its age range was broad, it cannot be stated that blind children between 6 and 7 years (our group average age) have a thicker cortex compared with an age-matched sighted population. Nevertheless, since the current perspective derived from adult studies, our data suggest that we may surely date back the onset of thickness changes in blind people at least around our group average age, although our thickness-by-age plots (Fig. 5) suggest that it might happen even earlier. Besides what happened in blind children cortex, the lack of a physiological thinning across age of the sighted 1 deserves a further comment. There is a general disagreement over cortex thickness evolution in children. While most of the first studies reported a U-shaped profile, with an increase in thickness until around 8–10 years of age (depending on the brain region) followed by a thinning (Sowell et al. 2004; Shaw et al. 2008; Raznahan et al. 2011), many recent papers reported instead a progressive thinning starting from 3 to 4 years of age (Brown and Jernigan 2012; Nguyen et al. 2013; Amlien et al. 2016). See Walhovd et al. (2017) for a more complete list of both kinds of studies. All these studies could investigate a huge cohort of subjects, which likely helped identifying significant correlations. We believe that we could not assess any significant relation between age and thickness (but in 1 cluster) because of the small size and large age distribution (10 years) of our sighted population.

The second result is that these differences emerged only when blindness was beyond 3 m (F-BS) or complete (T-BS). Brains of LV children, although their visual impairment was congenital, were not significantly different from a sighted brain. This implies that a congenital/early onset is a necessary condition for inducing thickness differences, but is not sufficient: A severe visual impairment is also needed to induce thickness changes. For completeness, uncorrected analysis suggested that LVS brain were closer, in term of thickness metric, to blind brain rather than sighted 1.

The third characteristic of the present thickening pattern is that it is multisystemic, also involving nonvisual brain areas, and changes in the occipital lobe are not located in V1, but rather in slightly more superior occipital regions (V2/V3) and are less extended than those previously found in adults. The latter differences can be, in our opinion, explained by our BS population. To allow statistical comparisons, we had to group together the total blind (T-BS) subjects with those blind beyond 3 m (F-BS). Assuming that lack of (visual) input is the main source of thickness deviation, the altered but still preserved inputs received by V1 synapses of F-BS should have granted their quite normal development and thus made BS group closer to NSC and LVS children. In this situation, it may have sense instead that a morphological alteration might have occurred in the associative visual areas of F-BS also. The cuneal areas hosting V2/V3, here found thicker, play an important role in more complex vision functions and might have been significantly altered, also in F-BS children, by the degraded level of their received input, and thus made significant the differences of the whole BS group.

### The Origin of Thickness Deviation

There is large consensus over the pivotal role of a reduced pruning mechanism in explaining the cortical thickening in visually deprived adults (Jiang et al. 2009; Park et al. 2009; Anurova et al. 2015; Li et al. 2017). Since focal thickening in visual areas have been associated to behavioral improvements in the acoustic domain (Voss and Zatorre 2012), a concurrent possible effect of cross-modal plasticity can be also expected. These phenomena would induce focal thickenings that partly counterbalance the generalized age-related thinning occurring also in adults blind brains (Anurova et al. 2015). In children, another physiological phenomenon has been recently suggested to alter the apparent CT derived from *T*_1_ MRI recordings. At least after 5 years old, a large part of the cortical thinning seems caused by the progressive myelination of the deep layers of the cortex. Myelination increases in fact *T*_1_-weighted voxel intensities and thus shifts the GM/WM interface towards the GM, reducing the apparent CT (Natu et al. 2019). Since visual losses due to disorders of the retina, as occurred in most of our subjects, are known to delay the myelination of visual system (Sonksen and Dale 2002), such delay could explain part of the thickening observed in blind children. Five years old is the age of their youngest participants, thus it cannot be excluded that this phenomenon may act even earlier.

The precocity of thickening and the lack of an evolution across time conflicts with the idea that pruning continues thinning the cortex until around either 5 (Johnson 1997) or 11 years (Huttenlocher and de Courten 1987). The different visual experience should in fact induce a different evolution of the thickness of the 2 populations until either 5 or 11 years, which here was not observed. On the other hand, cross-modal plasticity is expected as a late and progressive mechanism, associated to maturation and development and, although it cannot be excluded, seems unlikely to origin such an early age-independent thickening. The easiest explanation is that cortical pruning might progressively become less intense and thus its effect more difficult to be detected by the voxel resolution and magnetic field strength of the current *T*_1_ sequences. Alternatively, a very recent MRI paper, able to scan healthy children 7 times from 1 to 24 months (Wang et al. 2019), showed that cortex thickness peaked in average around 12–14 months, although not uniformly in all regions, and in most of them it maintained constant until 24 months. We can hypothesize that the genetically driven synaptogenesis could still be present after those peaks, but might have been initially counterbalanced by the beginning of the pruning mechanism. When synaptogenesis terminated and or pruning became more intense, thickness would have started to thin as largely reported (Brown and Jernigan 2012; Nguyen et al. 2013; Amlien et al. 2016). The reduced pruning in blind children instead would make the CT curve continue to grow and thus immediately start thickening the cortex of our subjects, being detectable also in our younger one. In both cases, the exact contribute of reduced pruning or delayed myelination shall be addressed by a proper multimodal approach (Walhovd et al. 2017; Natu et al. 2019).

The multisystemic nature of the present results deserves some comments. Although a similar multisystemic thickening pattern was also found in a recent work (Anurova et al. 2015), the absence of thickness modulation outside visual areas has been previously used (Jiang et al. 2009; Li et al. 2017) to support the pivotal role of reduced activity-dependent pruning in determining cortical thickening, making unlikely an effect of cross-modal plasticity. The present clusters of cortical thickening indicate instead a more complex pattern of multisystemic structural alterations. If, in adult studies, the inverse relationship between age and thickness (Anurova et al. 2015) might have thinned nonvisual areas, which are only indirectly and less massively affected by visual loss, our data reports instead brain changes occurring during a period of intense activity-dependent pruning and myelination, during which age did not have enough time to exert its effect. We believe that since pruning increases selectivity and effectiveness of synaptic activity and thus is synonymous of specialization and maturation (Rakic et al. 1986; Anurova et al. 2015), our thickening represents a marker of a delay in maturation of the resulting areas. This explanation fits particularly well with SPC thickening, being the latter areas densely connected with visual areas and fundamental for spatial functions (Husain and Nachev 2007) and cognition (Sack 2009), functions that can be highly impaired in blind people. Our ACC cluster overlaps with Brodmann areas a24 and p24 (Baker et al. 2018), which are both structurally connected with the posterior cingulate cortex until the posterior, visual-oriented, precuneus, whereas the latter is functionally connected to V1. More generally, the cingulate cortex is functionally implied in the processing of degraded images (Deary et al. 2004) and contributes, together with visual areas, to complement OFC in odor processing (Royet et al. 1999). Mesial precentral areas correspond to inferior limbs primary motor areas, cortical regions strictly involved in locomotion, a function that usually develops late and with reduced fluidity and variability of movement in blind children. Specific studies correlating possibly improved performance and thickness alteration in children are indeed needed to confirm that these thickenings are not a sort of compensatory changes, as once found in adults (Voss and Zatorre 2012). In most of adults studies, a minority of clusters resulted thinner in early blind with respect to sighted, whereas here we only found thicker region in blind. Those studies, most of which support the reduced pruning as the origin of the thicker cortex, simply reported such results without providing an explanation. We believe that their focal thinning could be related to complex plasticity mechanisms and different maturation processes occurred during later development, which in our population still did not take place.

Similar considerations could be raised to explain a partial inconsistency between the literature and the precocity of the present thickening. If thickening is such an early phenomenon, which mechanism is responsible for the tiny thickening observed in adolescent onset blindness previously reported? (Li et al. 2017). Again we can postulate that later plastic responses might induce the reported thickening in late blind. Besides the likely cross-modal plasticity previously reported (Voss and Zatorre 2012), we also know that visual cortex can, still in adulthood, undergo likely compensatory plasticity mechanisms. Central retinal lesions, typical of patients with macular degeneration, induce in fact a thinning of central responsive V1 regions and a thickening of the peripherally responsive ones (Burge et al. 2016) as a product of different visual strategies.

### Limitations

The present work has indeed some limitations, mainly related to being a retrospective study. Browsing the MRI database for a sufficient number of children fulfilling our strict clinical inclusion criteria (congenital VI children with peripheral only impairment), we had to use data obtained from 2 different MRI sequences, although recorded in the same scanner. We are aware that different recording parameters may alter the sensitivity of the reconstruction algorithms here employed and actually a trend toward a thicker cortex was found for sequence #2. For this reason, we opted to take into consideration and hence discuss only those clusters that resulted in both the analyses, the one considering all children (FULL) and the 1 only those recorded with sequence #2 (SEQ2). Nevertheless, we carefully attempted to manage the possible sequence bias to may include all the subjects. First, we validated each vertex toward a possible sequence effect within each group. All those vertices that were only liberally (not correcting for multiple comparisons) affected by the sequence effect were not reported in our results. Only the 13.38% of vertexes were marked as invalid and only 1 cluster, present in both FULL and SEQ2 analyses, was excluded for overlapping with the final results, which consequently look trustworthy. Second, we composed our NSC group preserving the same MRI sequences distribution (subjects with sequence #2 double than those with sequence #1) found in VI children. Accordingly, even assuming the presence of a subtle sequence-related thickening trend, since sequences distribution was preserved within all groups, such trend would have equally affected each group and thus should have not altered the present differences.

The included subjects’ number was rather low and forced us to merge data from a total and partial blindness. Even though they are quite different, they both share the impossibility to see farther than 3 m with a residual visus only for near distance in F-BS. From a functional perspective, they share a severe impairment that can lead to a significant lack of visual input in everyday activities. It is known that such visual impairment can affect the development of different functions such as spatial cognition (Pasqualotto and Proulx 2012), orientation skills, and mobility within the environment, and also the construction of one’s identity (Proulx et al. 2016). Unfortunately, behavioral and performance measures were not collected uniformly in all subjects and could not thus be correlated with children thickness profiles, which would have surely strengthened the significance of our findings.

## Conclusion

In the present study, we had the opportunity to investigate for the first time the effect of congenital blindness on the CT in children. According to our data, cortical thickening appears as a very early phenomenon, even much earlier than expected, as it established before the end of the pruning mechanism (either 5 or 11 years). We could also verify that this thickening occurred only when visual impairment induced not only a total blindness but also a minimal close-up visuals with no visual perception for far distance. Moreover, thickening was not limited to visual areas but extended to different brain regions, some of them implied in sustaining basic human activities, which may be limited or impaired in blind children, such as orientation in space and locomotion. Since cross-modal reorganization and age-related thinning are still to exert their effect fully, while thickening was present also in our younger subjects, we support that this thickening is due to a reduced pruning mechanism induced by visual deprivation and can be seen as a marker of reduced maturation of these regions.

## Supplementary Material

supplementary_materials_tgaa071

## Notes

The authors would like to acknowledge the precious contribution of the technicians of the Neuroradiology Department and the whole equipe of the Centre of Child Neuro-Ophthalmology of IRCCS Mondino Foundation in collecting the present data. *Conflict of Interest*: None declared.
