# Supplementary material for: Multisystemic Increment of Cortical Thickness in Congenital Blind Children
Source: Cereb Cortex Commun. 2020 Oct 9;1(1):tgaa071. doi: 10.1093/texcom/tgaa071 (PMC8152892; doi:10.1093/texcom/tgaa071)
Supplement: supplementary_materials_tgaa071 [file supplementary_materials_tgaa071.docx]

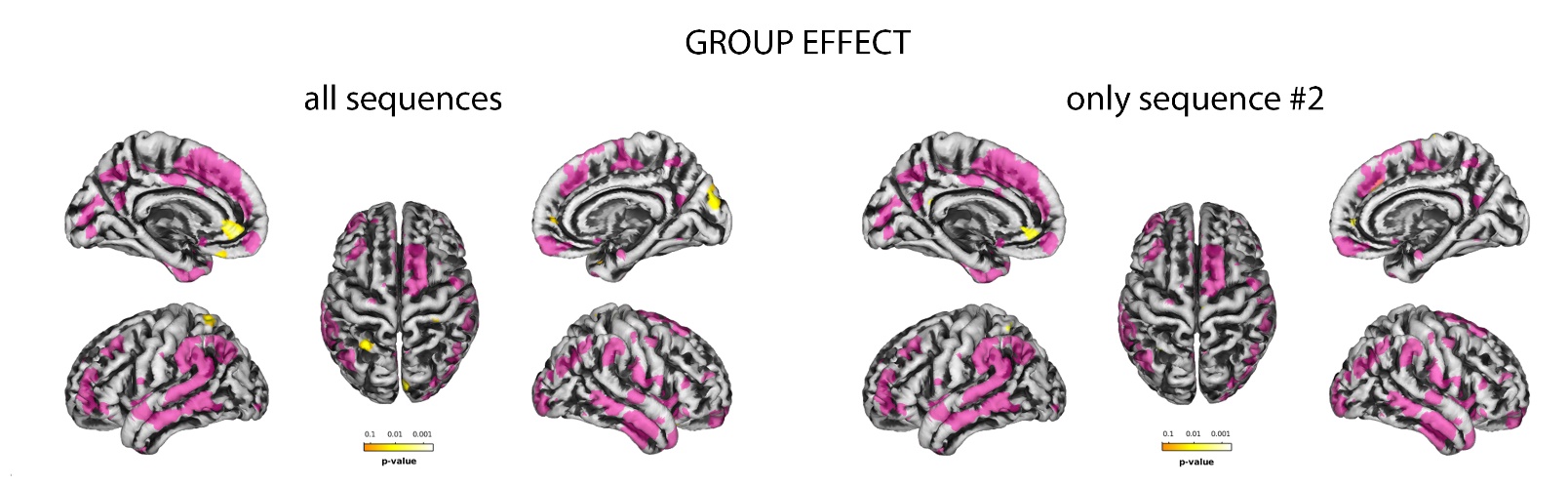


Figure 1S: Group main effect over cortical thickness, as revealed by One-Way ANOVA using age as covariate. Increased thickness in blind children with respect to sighted one. Results were FWE corrected, p<0.05. (left) Using all subjects, (right) using only subjects recorded with sequence #2. The exclusion mask is overlaid in semitransparent violet.

| Area | Cl. dim. | P | Z-score | x | y | z |
| --- | --- | --- | --- | --- | --- | --- |
| L ACC | 85 | 0.001 | 5.02 | -6 | 38 | -3 |
| R ACC | 25 | 0.013 | 4.45 | 6 | 49 | 4 |
| R OFC | 92 | < 0.001 | 4.78 | 27 | 19 | -24 |
| R cuneal cortex (V2/V3) | 38 | 0.007 | 4.66 | 6 | -85 | 28 |
| L SPC | 40 | 0.006 | 4.57 | -26 | -55 | 66 |
| R SPC | 25 | 0.017 | 4.53 | 18 | -48 | 70 |
| R lateral postcentral (S1) | 9 | 0.031 | 4.39 | 31 | -32 | 70 |
| R temporal pole | 7 | 0.035 | 4.24 | 28 | 11 | -31 |
| R ITC | 11 | 0.028 | 4.58 | 41 | -15 | -31 |

Table S1: Effect of group (FWE corrected One-Way Anova) over vertex-wise cortical thickness considering subjects recorded with both sequences. Abbreviations: Dim: number vertices belonging to the cluster, ACC: Anterior Cingulate Cortex, OFC: orbito-frontal cortex, SPC: superior parietal cortex, ITC: inferior temporal cortex.

| Area | Cl. dim. | P | Z-score | x | y | z |
| --- | --- | --- | --- | --- | --- | --- |
| L ACC | 35 | 0.004 | 4.44 | -3 | 40 | -8 |
| R ACC | 23 | 0.01 | 4.68 | 10 | 47 | 2 |
| L SPC | 12 | 0.022 | 4.61 | -31 | -56 | 56 |
| R mesial precentral (M1) | 5 | 0.047 | 4.28 | 5 | -21 | 78 |
| R ITC | 11 | 0.028 | 4.73 | 41 | -15 | -31 |

Table S2: Effect of group (FWE corrected One-Way Anova) over vertex-wise cortical thickness considering subjects recorded only with sequence #2. Abbreviations: Dim: number vertices belonging to the cluster, ACC: Anterior Cingulate Cortex, SPC: superior parietal cortex, ITC: inferior temporal cortex.


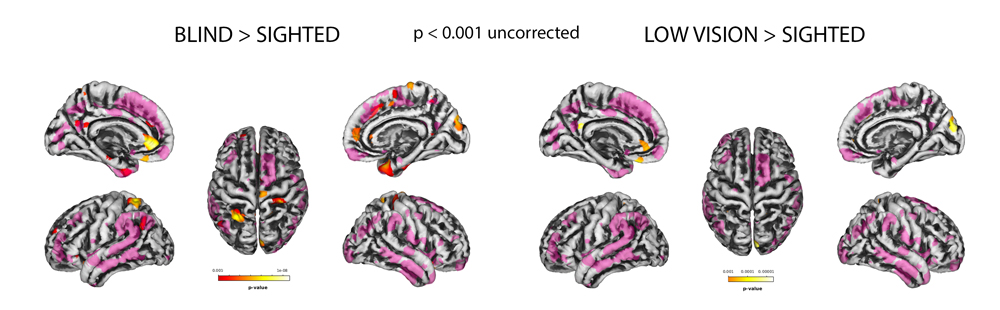


Figure S2: Uncorrected (p<0.001) group effect over cortical thickness, as revealed by two-samples t-test. Increased thickness in both blind and low vision children with respect to sighted one. The exclusion mask is overlaid in semitransparent violet.

| Cluster | Effect | Chi-square | p |
| --- | --- | --- | --- |
| Whole brain | group | 7.53 | 0.11 |
|  | age | 3.59 | 0.30 |
|  | group x age | 2.48 | 0.28 |
| L ACC | **group** | **18.55** | **0.0009** |
|  | age | 0.51 | 0.89 |
|  | group x age | 0.25 | 0.88 |
| R ACC | **group** | **22.05** | **0.0001** |
|  | age | 6.49 | 0.072 |
|  | group x age | 4.57 | 0.10 |
| R OFC | **group** | **14.56** | **0.0057** |
|  | age | 0.78 | 0.85 |
|  | group x age | 0.76 | 0.68 |
| R cuneal cx.(V2/V3) | **group** | **26.07** | **< 0.0001** |
|  | **age** | **18.55** | **0.0003** |
|  | **group x age** | **7.22** | **0.0271** |
| L SPC | **group** | **18.08** | **0.001** |
|  | age | 5.09 | 0.165 |
|  | group x age | 2.42 | 0.296 |
| R mesial precentral (M1) | **group** | **13.45** | **0.009** |
|  | age | 5.13 | 0.162 |
|  | group x age | 4.23 | 0.120 |

Table S3: ORL analysis of the mean CT in the six clusters affected by group factor and whole brain CT (mCT).
